# Microbial production of geranyllinalool at near 10 g per liter

**DOI:** 10.1186/s12934-026-03076-w

**Published:** 2026-08-18

**Authors:** Yi Zhou, Yuangen Lu, Jiongqin Liu, Jingde Qiu, Qiao Fan, Manxi Zhao, Xiao Ke, Hao Luo

**Affiliations:** 1 Sichuan Honghe biotechnology, Shuxi road 108, Chengdu, China; 2 Biotechnology Innovation Drug Application and Transformation Key Laboratory of Sichuan Province, Chengdu, China

**Keywords:** Geranyllinalool, Yeast, Fermentation, Metabolic, Synthetic

## Abstract

**Background:**

Acyclic diterpene alcohol, geranyllinalool (GL), is a natural insecticide and has diverse applications in cosmetics and pharmaceutical industries. The use of microbial cell factories for GL synthesis has been explored due to the non-sustainable nature of existing industrial manufacture methods.

**Results:**

This study focused on three technical challenges encountered in GL cell factory construction in *Saccharomyces cerevisiae*, namely cellular supply of geranylgeranyl pyrophosphate, selection of GL synthases, and decisive factors for large-scale bioprocess development. With design for industrial uses in mind, a robust yet efficient terpenoids-synthesis enabling chassis strain was acquired. Next, a bifunctional prenyltransferase/terpene synthase enzyme (GLS12) of high efficiencies was identified and characterized in detail. Inclusion of GLS12 in the terpenoids chassis resulted in ~ 0.8 g/L of GL synthesis in shake-flask cultivations. Scale-up study was completed with ~ 9 g/L GL produced in a 10-L bioreactor in 96 h by fed-batch fermentation, the highest reported titer to date.

**Conclusions:**

A high-performance yeast strain was engineered to enable efficient GL production using ethanol as the primary carbon source. This work demonstrated the potential of microbial GL fermentation for industrial uses.

**Graphical Abstract:**

**Figure:**
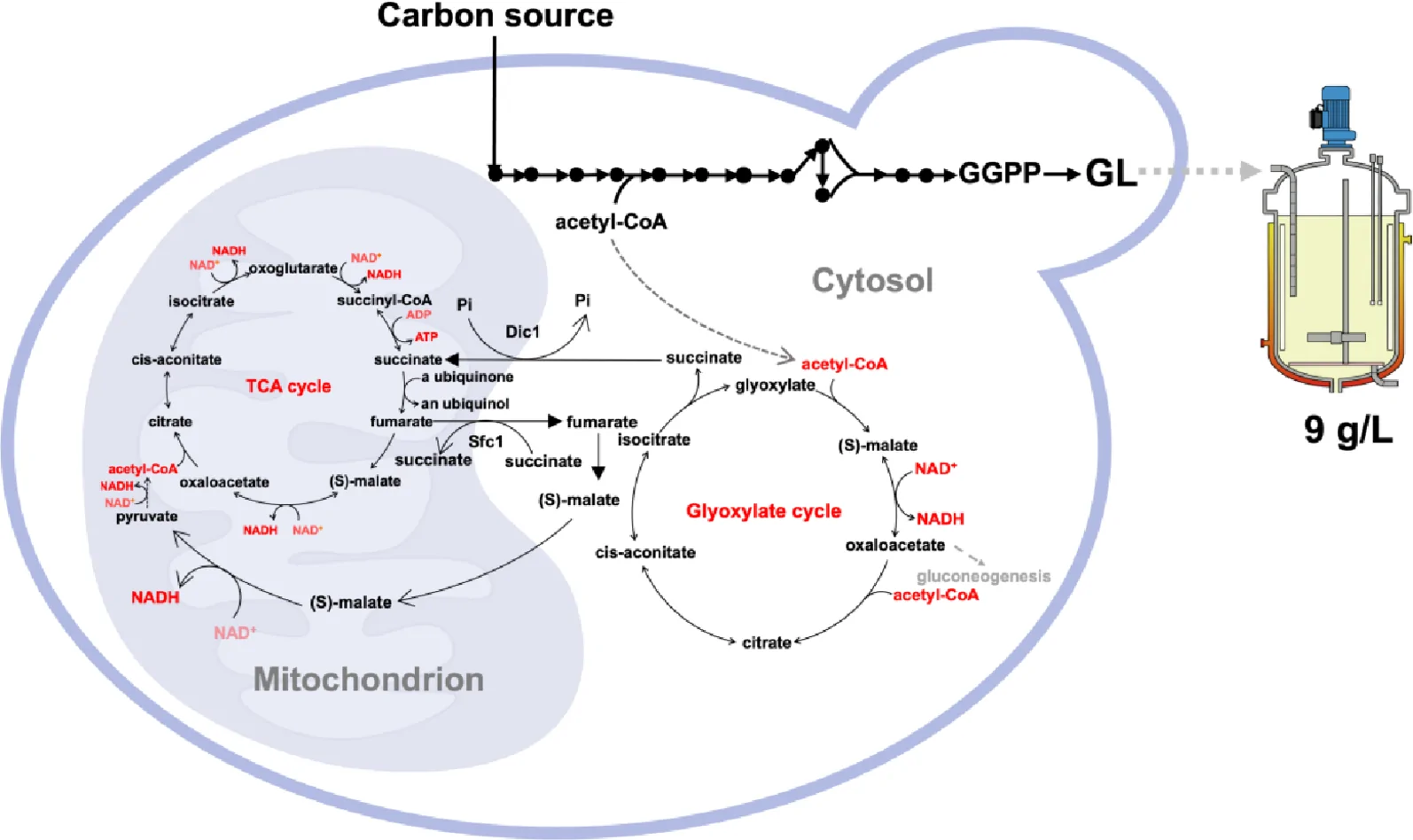

**Supplementary Information:**

The online version contains supplementary material available at https://doi.org/10.1186/s12934-026-03076-w.

## Background

Geranyllinalool (GL) is a naturally occurring acyclic diterpenoid of industrial interests. It is a natural insecticide for termites and certain ant predators [1]. It is a key intermediate to make teprenone, a drug used for acute gastroenteritis, exacerbated gastric inflammation, and gastric ulcers treatments [2]. Furthermore, it is extensively used as a fragrance ingredient in cosmetics, fine perfumes, shampoos, and household cleaning products [3]. However, existing GL manufacture relies on methods that are non-sustainable, for example plant extraction or chemical synthesis from petroleum derivatives, therefore bio-manufacturing using synthetic-biology tailored microbes is worthy of investigation [4].

Enzymatically, GL synthase (GLS) converts geranylgeranyl pyrophosphate (GGPP) to GL [5]. GLSs have been identified in several species including *Nicotiana attenuata*, *Tripterygium wilfordii*, and *Salvia guaranitica* [6–8]. Chang et al. (2024) [9] demonstrated it was feasible to make GL by heterologous expression of GLS from *N. attenuata* (NaGLS) via an isopentenol pathway in *Escherichia coli*. Despite their substantial engineering efforts, production levels of GL at 2 gram per liter remained economically non-feasible.

*Saccharomyces cerevisiae* is an industrially proven microbe to produce terpenoids. Notable examples are bioproduction of farnesene, squalene and sclareol [10–12]. The road-map for enabling terpenoids biosynthesis in *S. cerevisiae* is well described in the literature [13–15]. In this study, microbial production of GL in *S. cerevisiae* was explored via a combination of metabolic engineering and enzyme mining. To balance cost and productivity, the resulting strain was tailored to use ethanol for high-yield GL production. Last, scale-up potential of the final strain was demonstrated in a 10-liter bioreactor.

## Methods

### Strains and culture conditions

All strains and plasmids used in this study were available in supporting information (Table S1 and S2), including their definitive genotypes, promoters, terminators, and selection markers. All yeast expression plasmids used for yeast were of 2µ origin.

Engineered yeasts were made by homologous recombination using appropriate DNA cassettes. Briefly, the integration or expression cassettes, comprising the target genes driven by specific promoters and flanked by homologous arms and functional selection markers, were amplified from either the constructed recombinant plasmids or the *S. cerevisiae* genome using high-fidelity DNA polymerases. Yeast transformations were performed using the Frozen-EZ Yeast Transformation II Kit (Zymo Research, USA) according to the manufacturer’s instructions. Transformants were selected on appropriate synthetic defined (SD) dropout agar plates or rich media supplemented with antibiotics. The successful integration of genetic cassettes and plasmid adherence were verified by PCR and phenotype.

YPD and SD medium were used for routine yeast cultivations. F2 medium was used for all shake flask fermentations. The F2 medium was F1 with addition of 6 g/L succinate at pH 5.0. The composition of F1 medium was described by Zhang and coworkers [16]. Unless specified otherwise, G418 at 200 mg/L was supplemented when necessary.

### Shake flask fermentation

 For shake flask cultivations, pre-cultures were prepared in triplicates by growing a single colony in SD medium at 30℃ for 24 h. This was followed by diluting 1 mL pre-culture by 20 folds with F2 medium and cultivating at 30℃ until sampling. When required, ethanol was added to a concentration of 6 g/L.

### Fed batch fermentation

Fed-batch fermentations were performed using 3 L (CEKG, China) or 10 L (Sartorius) bioreactors. Per liter feed medium (I) contained 700 g glucose, 9 g KH_2_PO_4_, 5.125 g MgSO_4_.7H_2_O, 3.5 g K_2_SO_4_, 0.28 g Na_2_SO_4_, 10 mL trace metal solution, 12 mL vitamin solution, and 38.4 mg CaCl_2_.6H_2_O. The composition of trace metal and vitamin solutions was the same as F1 medium. Feed medium (II) was 80% ethanol. Up to 400 mL seed culture were prepared in F1 medium was inoculated into 3600 mL fermentation medium in the 10 L bioreactor. Up to 80 mL seed culture was inoculated into 720 mL fermentation medium in the 3 L bioreactor for fed-batch fermentation. The entire fermentation was run at 30℃, an agitation speed of 100–800 rpm, dissolved oxygen at above 30% and pH 5.0. Ammonia was used for pH adjustments. Feed medium (I) was added after ~ 24 h of fermentation. The cells were allowed to grow to an optical density (OD) of 150–230. This was followed by switching to Feed medium (II) with residual ethanol kept below 3 g/L. Samples were collected periodically. Measurements of glucose and ethanol were done using M-100 Bioanalyzer (Shenzhen Sieman Technology Co., Ltd.).

### Quantification of metabolites

Sample preparation was performed as follows. GL was extracted by vigorous mixing 0.75 mL fermentation broth with 0.3 mL n-dodecane and glass beads using a vortex. The dodecane layer was clarified by centrifuge and 0.1 mL sample was transferred to 0.9 mL of absolute alcohol for analysis. Squalene was extracted by mixing 0.5 mL fermentation broth with 0.5 mL ethyl acetate and glass beads. A programmed grinding at a speed of 10 m/s for 60 s and a 30 s pause was applied using a JXFSTRPR-40 grinder (Shanghai Jingxin, China). This process was repeated five times. The ethyl acetate layer was collected for analysis as above. MVA and malate were extracted by mixing 0.1mL of fermentation supernatant with 0.9 mL 8mM H_2_SO_4_ using a vortex.

GL was quantified using the Waters ACQUITY UPLC H-class equipped with an UV detector and a C18 column (Agilent Eclipse Plus RRHD C18, 150 mm×2.1 mm, 1.8 μm). GL was monitored at 208 nm. Separation (27 min) was performed using an acetonitrile/isopropanol (2:1) gradient with water as a diluent as following: 12 min at 80%, ramp to 100% in 1 min and hold for 10 min, reduce to 80% in 0.1 min and keep till the end. The flow rate was 0.2 mL/min. The column temperature was held at 30℃. Quantifications of squalene was the same as above in except the follows. Squalene was detected at 210 nm. Separation (16 min) was performed using an acetonitrile/ isopropanol (1:1) gradient with water as a diluent as following: 1 min at 95%, ramp to 100% and hold for 13 min, reduce to 95% in 0.1 min and hold for till the end of run. MVA and malate were quantified by HPLC following the procedure established by Niu and coworkers [17].

## Results and discussion

### The initial GL-producing strain

We started the design of GL strain by factoring in the use of ethanol at the beginning. In yeast, terpenoids biosynthesis via the mevalonate (MVA) pathway is proven to be highly efficient, but this pathway requires substantial cellular supply of acetyl-CoA (Fig. 1) [18]. When glucose is used as the carbon source, cytosolic acetyl-CoA of yeast is severely restricted. Much research has tried to address this scientific challenge [19–21] but all lead to significant host modifications, a major concern of strain stability. On the other hand, the simple solution from an industrial point of view is to use ethanol as the primary carbon source. This not only ascertains cytosolic acetyl-CoA sufficiency but also is economically operable at large scales. Although ATP usage to metabolize ethanol is substantial, provision of sufficient oxygen to engage oxidative phosphorylated ATP synthesis is plausible during fermentation through oxygenation or architectural improvements of the tanks.

**Fig. 1:**
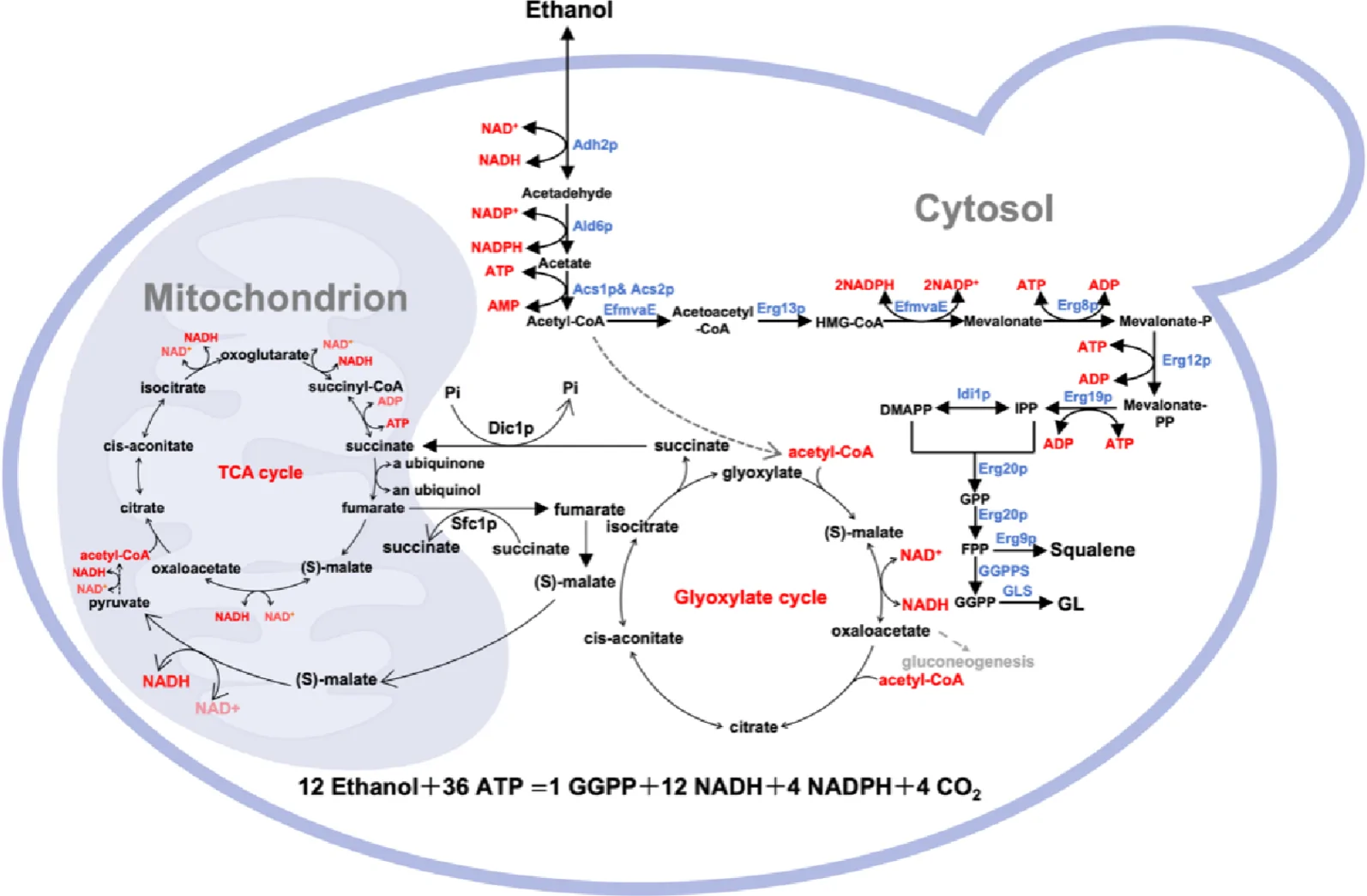
Drawing of GL biosynthesis pathways using ethanol. Ethanol is converted to cytosolic acetyl-CoA that is used for GL synthesis or energy metabolism. Abbreviations: DMAPP, dimethylallyl diphosphate; IPP, isopentenyl pyrophosphate; GPP, geranyl diphosphate; FPP, farnesyl diphosphate; TCA, tricarboxylic acid

To make the initial GL strain, two key enzymes, GGPP synthase (GGPPS) from *Blakeslea trispora* (BtCrtE) and NaGLS, were selected. BtCrtE was selected due to its demonstrated activity in engineering carotenoid-making yeast [22] and NaGLS was chosen because of its proven expression in *E. coli* [9]. An ethanol-inducible *ssa1* promoter, characterized previously [23], was used to regulate GLS expression while the expression of BtCrtE was driven by a constitutive *TEF1* promoter.

For initial host modifications, additional copies of the yeast MVA pathway biosynthetic genes (*ERG12* and *ERG13)* were introduced as well as the bifunctional acetyl-CoA acetyltransferase/HMG-CoA reductase from *E. faecalis* (*EfmvaE)* [24]. The expression of *ERG12* was driven by a *TEF1* promoter and *ERG13* and *EfmvaE* were expressed from the bi-directional *GAL1/GAL10* promoter. The combinatorial use of Δgal80 and a glucose-repressible *GAL1/GAL10* promoter was to accommodate sequential feeding of glucose and ethanol during fermentation. This allowed build up of the initial biomass while kept minimal *ERG13* and *EfmvaE* expression at the glucose feeding phase until transitioned to ethanol feeding to induce strong expression for production purposes.

The NADPH-dependent HMG-CoA reductase activity of *EfmvaE* was intentionally chosen. NADPH-dependent HMG-CoA reductase is considered as a rate-limiting step in MVA biosynthesis because the availability of NADPH is constrained under glucose-fed conditions [20]. When ethanol is used, a net of 4 molecules of NADPH (12 produced and 8 consumed) is produced in excess per GL produced. Thus, we reasoned the use of NADPH-consuming EfMvaE should favor in maintaining cytosolic NADP^+^/NADPH pool balance [25], establishing a driving force for terpenoids synthesis.

**Fig. 2:**
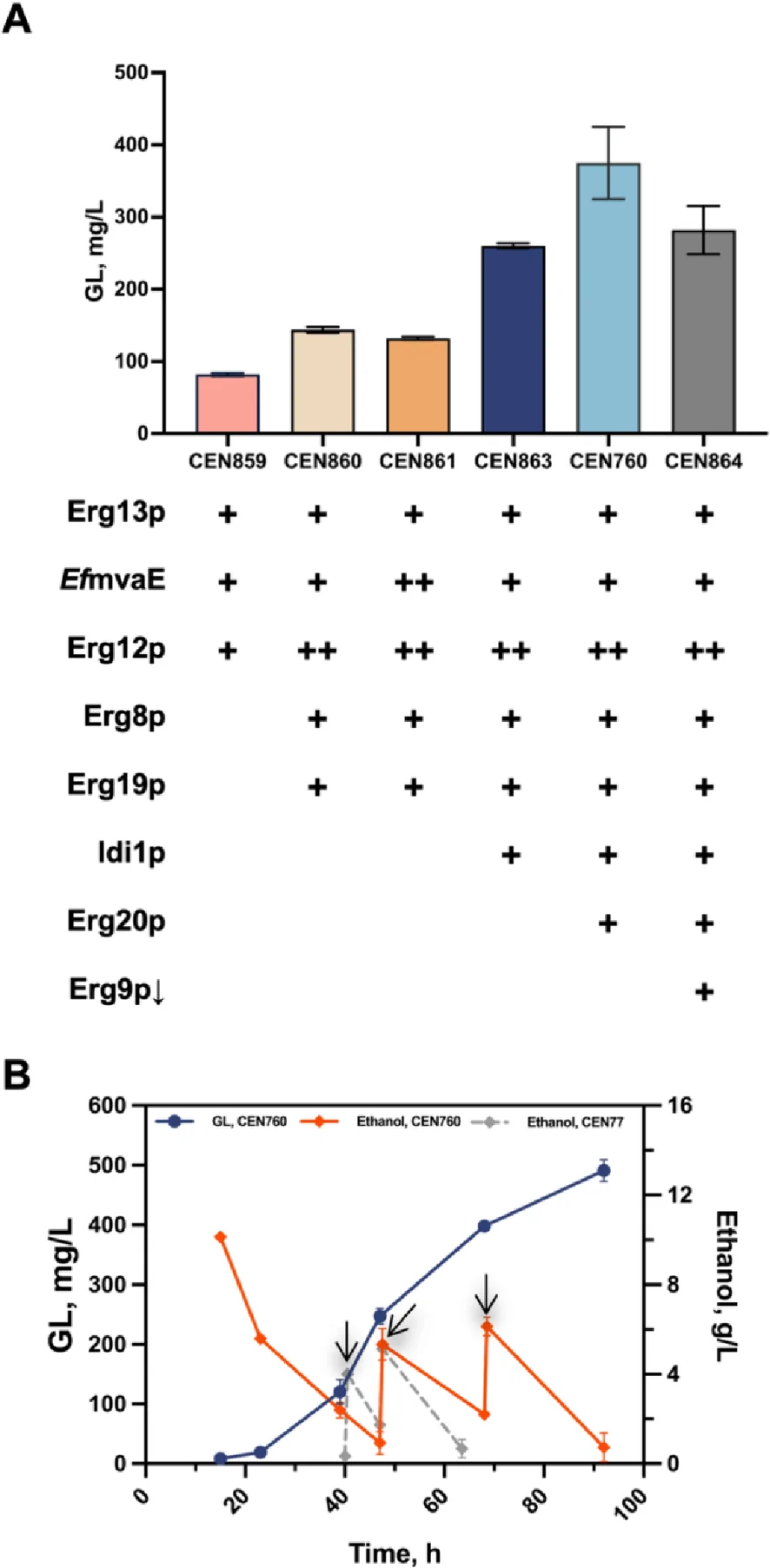
Construct and characterization of the initial GL-strain . **A** Iterative strain engineering of terpenoids chassis judged by GL production. Gene overexpression and copy number was marked with a plus sign (+), a downwards arrow (↓) indicated downregulation of a gene. **B** Performance of CEN760 in shake flasks with periodic ethanol feeding. Arrow indicated ethanol addition. Changes of GL and ethanol levels in CEN760 were shown in dark blue and orange lines while ethanol levels in wild type (CEN77) were shown in grey dashed line. Error bars, when shown, were calculated from at least 3 biological replicates

The initial GL-producing or the benchmark strain was designed as CEN859. In shake-flask cultivations, this strain was capable of 82 ± 2 mg/L GL synthesis (Fig. 2A). Further production increase was achieved by incorporating additional copies of *ERG12*, *ERG8*, and *ERG19* to promote terpenoids precursor isopentenyl pyrophosphate (IPP) synthesis. This resulted in 143 ± 4 mg/L GL production (CEN860), an increase of ~ 74% compared to benchmark (Fig. 2A). Noted, two copies of *ERG12* had to be introduced to reduce extracellular MVA overflow (data not shown). This suggested CEN860 carried high levels of MVA synthesis and supported the assumed NADP^+^/NADPH balance driven synthesis via EfMvaE. Furthermore, expression of an additional copy of *EfmvaE* (CEN861) did not show significant titer improvement, hinting limitations of downstream pathway enzymes (Fig. 2A).

Indeed, incorporating an extra copy of the *IDI1* gene further improved GL synthesis. The resulting strain CEN863 produced 260 ± 3.5 mg/L of GL (Fig. 2A). This result confirmed Idi1, which catalyzes IPP-DMAPP interconversion, was rate-limited in CEN860. Lastly, combinational expression of *IDI1* and *ERG20* doubled GL production to 375 ± 50 mg/L (Fig. 2A) in CEN760. This was in line with expectations since *ERG20* overexpression was known to improve the abundance of farnesyl pyrophosphate (FPP) needed for downstream synthesis of GGPP and GL.

Worth noting that reduction of squalene synthase expression had a negative impact on GL production. Squalene synthase encoded by *ERG9*, which is essential for yeast growth [26], was a competitor of BtCrtE for FPP. Downregulation of *ERG9* by replacing its native promoter with a *GAS3* promoter, which was a weaker promoter than one of *ERG9* in the presence of ethanol according to the transcriptomic data [27], was made to further improve GGPP synthesis. The resulting strain (CEN864) showed a decrease in final GL production to 281 ± 33 mg/L (Fig. 2A). This suggested manipulation of *ERG9* expression using a *GAS3* promoter was not desirable.

Next, we assessed the suitability of CEN760 for scale-up studies. This strain was studied in shake flasks with periodic ethanol feeding to mimic fed-batch fermentation. Ethanol and GL levels were monitored in time series. As shown in Fig. 2B, a peak titer of 495 ± 18 mg/L was obtained at 92 h after two constitutive ethanol feeds. Despite continuous synthesis, GL production deteriorated over time accompanying by a worrisome ethanol consumption rate which was noticeably slow in comparison to wild type. Together, both metrics indicated CEN760 was not suitable for scaling up and forced us to withdraw further developments on this strain.

## Enzyme mining and identification of GLS12

Based on the results above, we started to mine additional enzyme candidates. Three GGPPSs from *Pantoea agglomerans* [28], *Taxus x media* [29], Erg20p(F96C) of *S. cerevisiae* [30] were selected as BtCrtE replacements. When compared, none outperformed BtCrtE (Fig. 3A). In the selection of GLSs, we firstly had to create a BtCrtE-stable expression strain (CEN702) because plasmids bearing *BtCrtE* were hard to obtain. CEN702 carried a genomic insertion of *BtcrtE* in CEN2022, the host strain of CEN760. GLSs from *Capsicum annuum* (CaGLS), *Theobroma cacao* (TcGLS), *Actinidia chinensis* var. *Chinensis* (AcGLS), and *Solanum commersonii* (ScGLS) were introduced into CEN702 and were assessed against CEN751, which was CEN702 expressing NaGLS. As shown in Fig. 3B, NaGLS had higher activity than all newly identified GLSs.

**Fig. 3:**
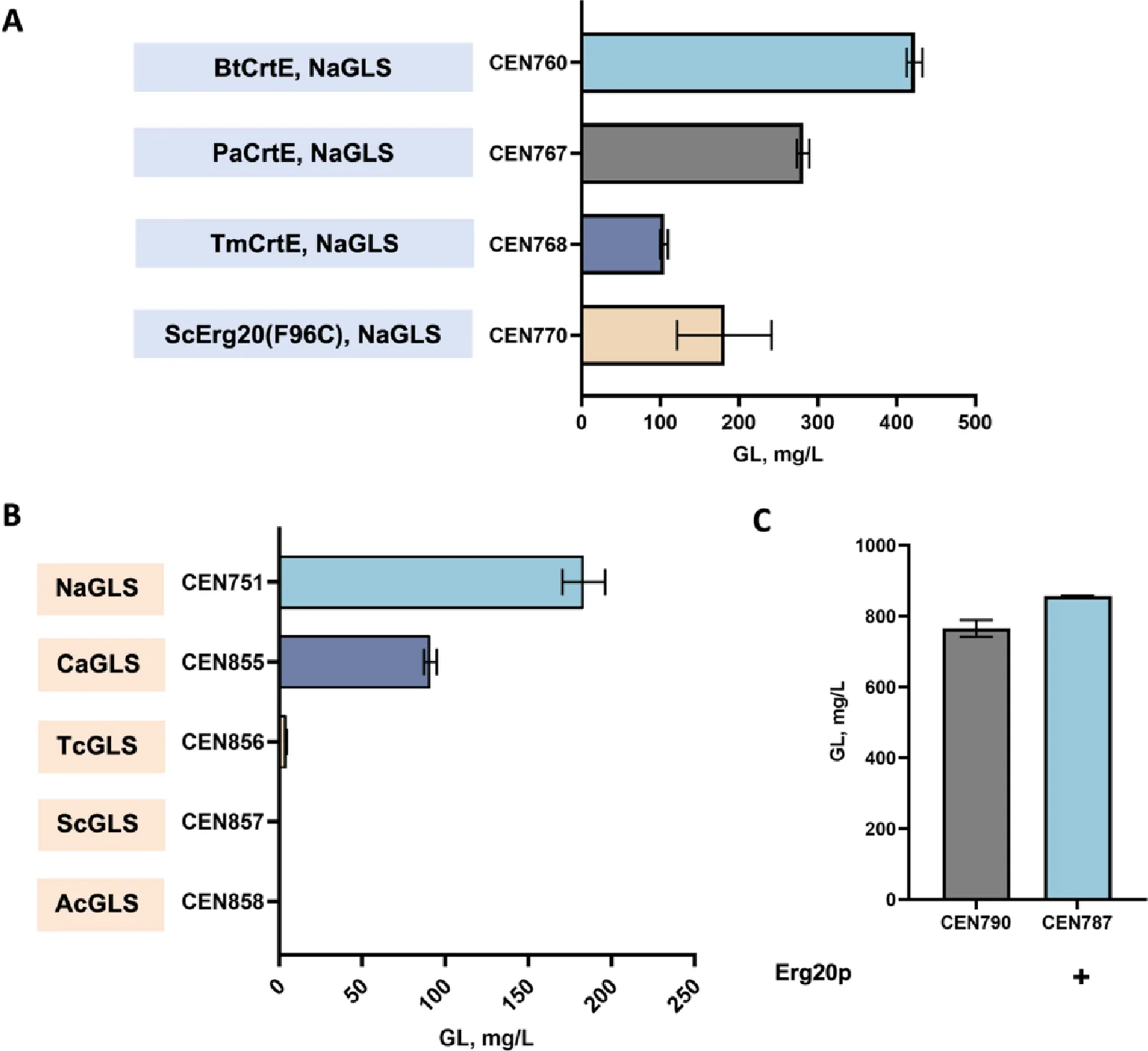
Bioprospecting of GGPPs and GLSs. Production performance of different GGPPS (**A**) and GLS (**B**) strains in shake flasks. Shake-flask production of GLS12 in strains with *ERG20* (-/+) overexpression (**C**). Error bars, when shown, were calculated from at least 3 biological replicates

Being unable to identify superior GGPPSs and GLSs, bioprospecting was expanded to naturally occurring bifunctional chimeric enzymes. Chen et al. [31] reported PTTS013 or named GLS12 from *Aspergillus niger* was able to make GL in *S. cerevisiae* albeit unknown efficiencies. Inspired by the work, we examined GLS12 in CEN2021 and CEN2022 creating CEN790 and CEN787, respectively. The CEN2021 resembled CEN2022 but lacked *ERG20* overexpression. It was used because of possible alternative substrate preferences of GLS12 as suggested by Chen and co-authors [31].

Results showed both strains outperformed CEN760. CEN790 produced 752 ± 24 mg/L GL in flask cultivations, ~ 2 folds that of CEN760 (Fig. 3C). In comparison, the CEN787 strain produced a titer of 857 ± 1 mg/L, ~ 14% higher than that of CEN790, concluding GLS12 was not only highly active in yeast but preferred FPP to use in vivo.

## Scaling up study

Lastly, we tried to scale up GL production in bioreactors. Since commercial GL production was intended, we created CEN786 by introducing the GLS12-bearing plasmid of above into CEN2039, which resembled CEN2022 genotype and complied to the terms of commercial uses (Table S1). First, CEN786 was assessed using shake-flask ethanol feeding tests. In contrast to CEN760, cells maintained near linear GL production reaching as high as 4 g/L at the end of cultivation (Fig. 4A). Within 64 h, ethanol was fed 3 times and was fully consumed suggesting robust ethanol consumption and readiness for bioreactor studies.

Subsequently, fed-batch fermentations were performed using a 10-L bioreactor. Changes in GL, ethanol and OD were shown in Fig. 4B. In summary, a maximum GL titer of ~ 9 g/L and a yield of 7.7% g/g (0.012% mol/mol) was obtained at the end of 96 h fermentation. A maximum production rate of 0.43 g/L/h occurred at 39–47 h just after switching to ethanol feeding and the max OD was at ~ 400. Peak GL production titer was recorded at ~ 70 h and did not increase further till the end of run. Similar trends were observed in multiple runs (Figure S1).

The loss of momentum in GL production during fermentation was puzzling. This was because cells were metabolically active as evidence by continuing growth of additional 100 OD. Ability of CEN2039 in providing sufficient precursors for terpene synthesis was then suspected. It prompted us to benchmark CEN2039 by measuring its ability making squalene, an indigenous molecule derived from FPP. As shown in Fig. 4C, CEN2701, which was CEN2039 carrying *ERG9* overexpression, was able to accommodate up to 46 g/L of squalene under a comparable fermentation process as that of the GL strain. The calculated yield was 18.2% g/g (0.02% mol/mol) that was significantly higher than the one obtained for the GL strain eliminating our doubts on CEN2039. At this stage, the exact cause led to GL production halt was not identified. Elevated ethanol started to accumulate after GL reached 5 g/L or above but did not occur in making of squalene and it was possible high concentrations of GL induced inhibition of certain enzymes.

**Fig. 4:**
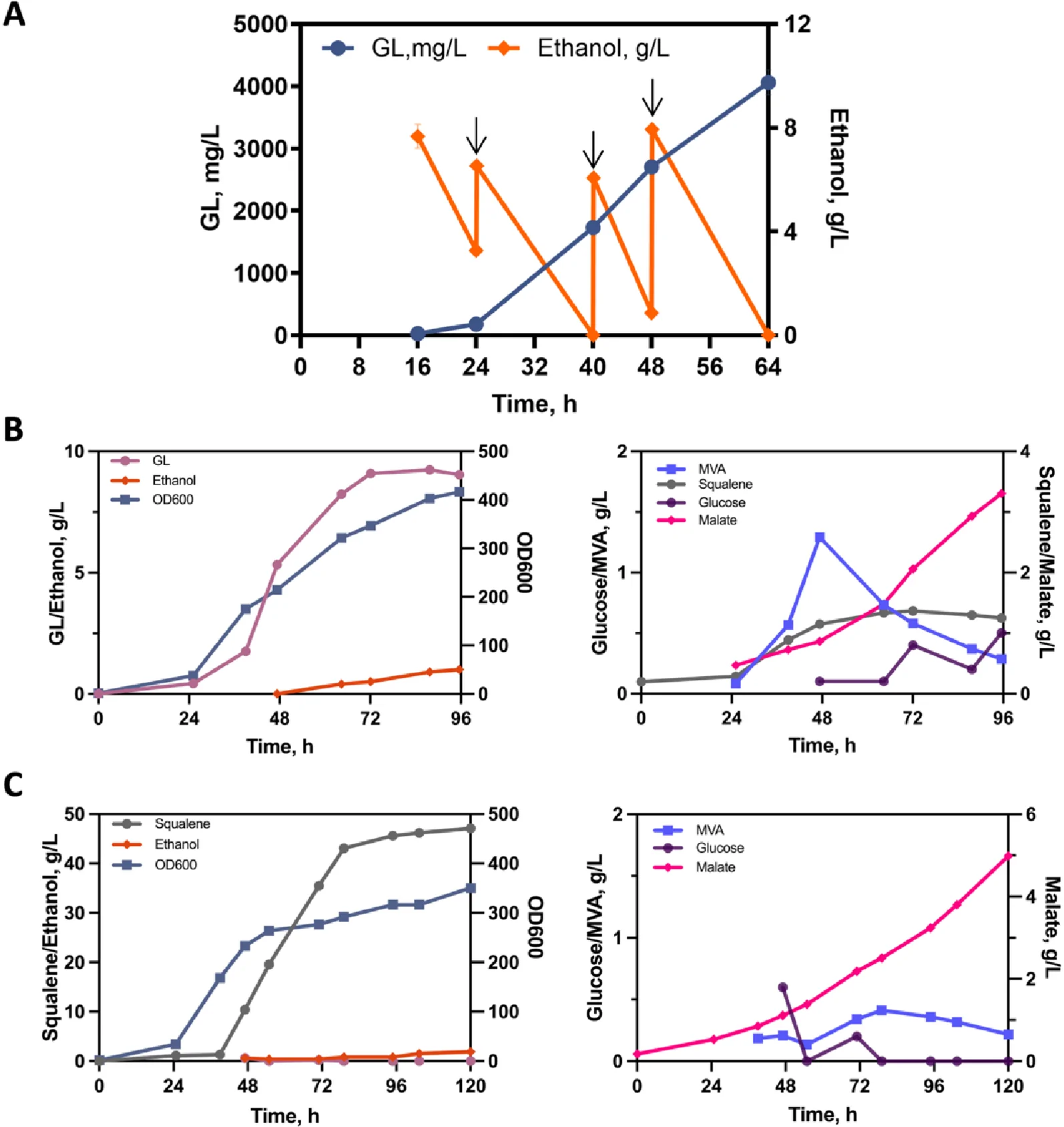
Study of GLS12-bearing strain for industrial uses. **A**. Performance of CEN786 in shake flasks with periodic ethanol feeding. Arrows indicated time points of ethanol additions. Changes of GL and ethanol levels were shown in dark blue and orange lines. Representative fed-batch fermentation processes of GL-producing strain CEN786 (**B**) and squalene-producing strain CEN2701 (**C**) in bioreactors. Changes of GL, squalene, optical density at 600 nm (OD600), ethanol, glucose, MVA and malate were shown in purple, dark grey, dark blue, orange, dark violet and light blue and pink lines. Error bars, when shown, were calculated from at least 3 biological replicates

## Conclusions

In this study, we successfully constructed a GL-producing yeast strain capable of near 10 g/L. The strain was designed and optimized for the use of ethanol as the primary carbon source. The production titer of this strain was the highest reported value to date and ~ 4 folds higher than the one previously reported in *E. coli* [9]. A techno-economical analysis showed the yeast-based bioprocess developed in this study was significantly more cost-efficient than the one employing *E. coli* (Table 1). Together, our results supported the use of an ethanol-based yeast bioprocess as an economically viable option for bioproduction of GL. This study set a solid foundation towards industrial GL production.

**Table 1:** Cost comparison of producing one kilogram of GL using *E.coli* or yeast

	Chang et.al(2024)	This study
Organism	*E.coli*	Yeast
Titer (g/L)	2.06	9
Bioreactor time (h)	150	96
Carbon source	Glycerol	Glucose/ethanol
Supplements	Prenol	None
Yield (%, g/g)	32.6	7.7
	Cost (US$)for per kilo of GL	
Fermentaton broth(a)	$1,733.00	$397.00
Supplements	$163.00	0
Power consumption	Not calculated	Not calculated
Total	$1,896.00	$397.00

(a) Assumed cost for defined minimal medium is at $3.5 per liter

## Supplementary Information

Below is the link to the electronic supplementary material.

Supplementary Material 1. Figure S1: Fed-batch fermentation of CEN786 in 10-L bioreactor (A) and 3-L bioreactors (B, C). Table S1: Yeast strains used in this study. Table S2: Plasmids used in this study. Supplementary Sequences.

## Data Availability

All data generated or analyzed during this study are included in this published article and its supplementary information files.

## Abbreviations

GL: Geranyllinalool
DMAPP: Dimethylallyl diphosphate
IPP: Isopentenyl pyrophosphate
GPP: Geranyl diphosphate
FPP: Farnesyl diphosphate
GGPP: Geranylgeranyl pyrophosphate
TCA: Tricarboxylic acid
MVA: Mevalonate
*S. cerevisiae*: *Saccharomyces cerevisiae*
*E. coli*: *Escherichia coli*
