# Supplementary material for: Microbial production of geranyllinalool at near 10 g per liter
Source: Microb Cell Fact. 2026 Aug 18;25:239. doi: 10.1186/s12934-026-03076-w (PMC13760993; doi:10.1186/s12934-026-03076-w)
Supplement: Supplementary file 1 [file 12934_2026_3076_MOESM1_ESM.docx]

**Supporting information: Microbial production of geranyllinalool at near 10 g per liter**

**Figure S1**  Fed-batch fermentation of CEN786 in 10-L bioreactor (A) and 3-L bioreactors (B, C).

| **Table S1: Yeast strains used in this study** | | |
| --- | --- | --- |
| **Strains** | **Genotype** | **Sources** |
| CEN.PK2-1D | MATα,*ura3-52;trp1-289;leu2-3,112;his3Δ1; MAL2-8c; SUC2* | European Saccharomyces cerevisiae Archive for Functional Analysis（EUROSCARF） |
| CEN77 | CEN.PK2-1D;*ura3-52::URA3;trp1-289::TRP1; leu2-3,112::LEU2; his3Δ1::HIS3* | This study  Selection markers were completely restored. |
| CEN2001 | CEN.PK2-1D;Δ*gal80*::P*his3*-*HIS3*-P*tef1*-*ERG12*-Terg12-T*erg13*(RC)-*ERG13(*RC)-P*gal1*(RC)-P*gal1*0-*Ef*mvaE-T*gal80* | This study |
| CEN850 | CEN2001;XI-3::P*trp1*-*TRP11*-T*trp1* | This study |
| CEN851 | CEN850;Δ*lpp1*::P*ura3-URA3*-T*ura3* | This study |
| CEN859 | CEN851;pL2μ-T*cyc1*(RC)-*BtcrtE*(RC)-P*tef1*(RC)-P*ssa1*-*NaGLS*-T*adh1* | This study |
| CEN2003 | CEN2001;XI-3::P*trp1*-*TRP1*-T*trp1*-P*tef1*-*ERG12*-T*erg12*-P*fba1*-*ERG8*-T*erg8*-P*tef2*-*ERG19*-T*erg19* | This study |
| CEN2012 | CEN2003;Δ*lpp1*::P*ura3-URA3*-T*ura3* | This study |
| CEN860 | CEN2012;pL2μ-T*cyc1*(RC)-*BtcrtE*(RC)-P*tef1*(RC)-P*ssa1*-*NaGLS*-T*adh1* | This study |
| CEN2007 | CEN2003;416d::P*ura3*-*URA3*-T*ura3*-P*tef1*-*Ef*mvaE-T*pgi1* | This study |
| CEN861 | CEN2007;pL2μ-T*cyc1*-*BtcrtE*(RC)-P*tef1*-P*ssa1*-*NaGLS*-T*adh1* | This study |
| CEN2021 | CEN2003;Δ*lpp1*::P*ura3-URA3*-T*ura3*-P*tef2*-*IDI1*-T*idi1* | This study |
| CEN863 | CEN2021;pL2μ-T*cyc1*(RC)-*BtcrtE*(RC)-P*tef1*(RC)-P*ssa1*-*NaGLS*-T*adh1* | This study |
| CEN2022 | CEN2003;Δ*lpp1*::P*ura3*-*URA3*-T*ura3*-P*tef1*-*ERG20*-T*cyc1*-P*tef2*-*IDI1*-T*idi1* | This study |
| CEN760 | CEN2022;pL2μ-T*cyc1*(RC)-*BtcrtE*(RC)-P*tef1*(RC)-P*ssa1*-*NaGLS*-T*adh1* | This study |
| CEN2044 | CEN2003;ΔP*erg9*::P*gas3*-*ERG9-*P*ura3*-*URA3*-T*ura3*-P*tef1*-*ERG20*-T*cyc1*-P*tef2*-*IDI1*-T*idi1* | This study |
| CEN864 | CEN2044;pL2μ-T*cyc1*(RC)-*BtcrtE*(RC)-P*tef1*(RC)-P*ssa1*-*NaGLS*-T*adh1* | This study |
| CEN767 | CEN2022;pL2μ-T*cyc1*(RC)-*PacrtE*(RC)-P*tef1*(RC)-P*ssa1*-*NaGLS*-T*adh1* | This study |
| CEN768 | CEN2022;pL2μ-T*cyc1*(RC)-*TmcrtE*(RC)-P*tef1*(RC)-P*ssa1*-*NaGLS*-T*adh1* | This study |
| CEN770 | CEN2022;pL2μ-T*cyc1*(RC)-*ScERG20*(F96C)(RC)-P*tef1*(RC)-P*ssa1*-*NaGLS*-T*adh1* | This study |
| CEN702 | CEN2022;XII1::P*tef1*-*BtcrtE*-T*cyc1*-KanMX | This study |
| CEN751 | CEN702;pL2μ-P*ssa1*-*NaGLS*-T*adh1* | This study |
| CEN855 | CEN702;pL2μ-P*ssa1*-*CaGLS*-T*adh1* | This study |
| CEN856 | CEN702;pL2μ-P*ssa1*-*TcGLS*-T*adh1* | This study |
| CEN857 | CEN702;pL2μ-P*ssa1*-*ScGLS*-T*adh1* | This study |
| CEN858 | CEN702;pL2μ-P*ssa1*-*AcGLS*-T*adh1* | This study |
| CEN790 | CEN2021;pL2μ-P*ssa1*-GLS12-T*adh1* | This study |
| CEN787 | CEN2022;pL2μ-P*ssa1*-GLS12-T*adh1* | This study |
| CEN2037 | CEN.PK2-1D;XI3::P*trp1*-*TRP1*-T*trp1*-P*tef1*-*ERG12*-T*erg12*-P*fba1*-*ERG8*-T*erg8*-P*tef2*-*ERG19-*T*erg19* | This study  To comply the terms for commercial use, promoter and terminator DNA sequences used for genetic manipulations were all derived from CEN.PK2-1D. |
| CEN2038 | CEN2037;Δ*gal80*::P*his3*-*HIS3*-T*his3*-P*tef1*-*ERG12*-T*erg12*-T*erg13*(RC)-*ERG13*(RC)-P*gal1*(RC)-P*gal1*0-*Ef*mvaE-T*gal80* | This study  To comply the terms for commercial use, promoter and terminator DNA sequences used for genetic manipulations were all derived from CEN.PK2-1D. |
| CEN2039 | CEN2038;*Δlpp1*::P*ura3*-*URA3*-T*ura3*-P*tef1*-*ERG20*-T*adh1*-P*tef2*-*IDI1*-T*idi1* | This study  To comply the terms for commercial use, promoter and terminator DNA sequences used for genetic manipulations were all derived from CEN.PK2-1D. |
| CEN786 | CEN2039;pL2μ-P*ssa1*-GLS12-T*adh1* | This study |
| CEN2701 | CEN2039;pL2μ-P*ssa1*-*ERG9*-T*adh1* | This study |
| Remarks：P, promoter；T, terminator. | | |

| **Table S2: Plasmids used in this study** | | |
| --- | --- | --- |
| **Plasmids** | **Sources** | **Remarks** |
| pL2μ | Lab stock | LEU2 AmpR ColE1 2μ |
| pL2μ-T*cyc1*(RC)-*BtcrtE*(RC)-P*tef1*(RC)-P*ssa1*-*NaGLS*-T*adh1* | This study | *BtcrtE* and *NaGLS* gene synthesized |
| pL2μ-T*cyc1*(RC)*-PacrtE*(RC)-P*tef1*(RC)-P*ssa1*-*NaGLS*-T*adh1* | This study | *PacrtE* gene synthesized |
| pL2μ-T*cyc1*(RC)-*TmcrtE*(RC)-P*tef1*(RC)-P*ssa1*-*NaGLS*-T*adh1* | This study | *TmcrtE* gene synthesized |
| pL2μ-T*cyc1*(RC)-*ScERG20*(F96C)(RC)-P*tef1*(RC)-P*ssa1*-*NaGLS*-T*adh1* | This study |  |
| pL2μ-P*ssa1*-*NaGLS*-T*adh1* | This study | gene synthesized |
| pL2μ-P*ssa1*-*AcGLS*-T*adh1* | This study | Uniprot ID:A0A2R6RAS9  gene synthesized |
| pL2μ-P*ssa1*-*ScGLS*-T*adh1* | This study | Uniprot ID:A0A9J5ZPH1  gene synthesized |
| pL2μ-P*ssa1*-*TcGLS*-T*adh1* | This study | Uniprot ID:A0A061GFZ6  gene synthesized |
| pL2μ-P*ssa1*-*CaGLS*-T*adh1* | This study | Uniprot ID:A0A2G2Y4L3  gene synthesized |
| pL2μ-P*ssa1*-GLS12-T*adh1* | This study | Uniprot ID:A0A100IAT3  gene synthesized |
| pL2μ-P*ssa1*-*ERG9*-T*adh1* | This study |  |
| Remarks：P, promoter；T, terminator. |  |  |

**Supplementary Sequences:**

**Promoters**

>*ssa1*

atagtgggtagataaccttaaactatttatttagaggcgacaatgtgaaaaattttttcttcaaaggctcggttgtcgacaaattgttacgttgtgctttgatttctaaagcgcttcttcacctgcaggttctgagccctaagaaaaaaaatttccttggttgaaaatggcggaaaaaaaaaattcagaaaaagaaataaagcacgtgtgcgcggtgtgtggatgatggtttcatcattgtcaacggcattttcgttcttgtggattgttgtaaactttccagaacattctagaaagaaagcacacggaacgtttagaagctgtcatttgcgttttttctccagattttagttgagaaagtaattaaattattcttctttttccagaacgttccatcggcggcaaaagggagagaaagaacccaaaaagaaggggggccatttagattagctgatcgtttcgaggacttcaaggttatataaggggtggattgatgtatcttcgagaagggattgagttgtagtttcgtttcccaattcttacttaagttgttttattttctctatttgtaagataagcacatcaaaagaaaagtaatcaagtattacaagaaacaaaaattcaagtaaataacagataat

*>tef1*

agtgatcccccacacaccatagcttcaaaatgtttctactccttttttactcttccagattttctcggactccgcgcatcgccgtaccacttcaaaacacccaagcacagcatactaaatttcccctctttcttcctctagggtgtcgttaattacccgtactaaaggtttggaaaagaaaaaagagaccgcctcgtttctttttcttcgtcgaaaaaggcaataaaaatttttatcacgtttctttttcttgaaaatttttttttttgatttttttctctttcgatgacctcccattgatatttaagttaataaacggtcttcaatttctcaagtttcagtttcatttttcttgttctattacaactttttttacttcttgctcattagaaagaaagcatagcaatctaatctaagttttaattacaaa

**Terminators**

>*adh1*

agttataaaaaaaataagtgtatacaaattttaaagtgactcttaggttttaaaacgaaaattcttattcttgagtaactctttcctgtaggtcaggttgctttctcaggtatagcatgaggtcgctcttattgaccacacctctaccggcatgccga

*>cyc1*

atccgctctaaccgaaaaggaaggagttagacaacctgaagtctaggtccctatttatttttttatagttatgttagtattaagaacgttatttatatttcaaatttttcttttttttctgtacagacgcgtgtacgcatgtaacattatactgaaaaccttgcttgagaaggttttgggacgctcgaag

**Genes**

>*BtcrtE*

atgttgacttcttctaaatctattgaatcttttccaaaaaatgttcaaccatatggtaagcattatcaaaatggtttggagccagtcggtaagtctcaggaggacattttgttagagccattccactacttgtgctctaaccctggtaaggacgtcaggactaagatgatagaagcattcaatgcatggttgaaggtcccaaaggacgatttgatagttattacaagggttattgagatgttgcactctgcatcattattgattgacgacgtcgaggacgactcagtcttgaggagaggtgtccctgctgcacaccacatttacggaacaccacagactataaactgtgctaattacgtctacttcttggctttaaaggagattgctaagttaaacaaacctaacatgattactatttacacagacgagttgattaacttgcacaggggtcagggtatggagttgttctggagggacacattgacttgcccaacagagaaggagttcttggacatggtcaatgacaaaactggtggattgttgaggttggctgtcaagttgatgcaggaggcttctcagtcaggaactgactacacaggattggtttcaaagattggaatacatttccaggtcagggacgattatatgaatttacagtcaaaaaactacgcagacaacaagggattctgcgaggacttgacagagggaaagttctctttcccaataatacactcaattaggtctgacccatcaaacaggcagttgttgaacatattgaaacagagatcttcatctattgaattgaaacaattcgcattgcagttattggaaaatacaaacactttccaatattgcagagacttcttgagggtcttggagaaggaggcaagagaggaaattaaattgttgggtggtaatataatgttagaaaagattatggacgtcttgtctgtcaacgaataa

>*PacrtE*

atggtttctggttctaaggctggtgtctcaccacacagggagattgaggtcatgaggcagtctattgacgatcacttggctggtttgttgcctgagactgactctcaggacattgtctcattggcaatgagggagggtgtcatggctccaggtaagaggataaggcctttgttgatgttgttggcagctagggacttgaggtaccagggttctatgcctactttgttggacttggcttgcgctgtcgaattgactcacactgcatcattgatgttggacgacatgccttgcatggacaacgcagaattaaggaggggtcagccaacaacacacaagaagttcggtgagtcagtcgcaattttggcatcagttggtttgttatcaaaggctttcggattgattgctgcaactggtgacttaccaggtgagaggagggcacaggctgtcaacgagttgtctactgctgtcggagtccagggattggtcttgggtcagttcagggacttgaacgacgcagctttggacaggactccagacgctatattgtctacaaaccacttaaagacaggaattttgttctctgctatgttgcagatagtcgctattgcatctgcttcttctccatctacaagggagactttgcacgctttcgcattggacttcggtcaggctttccagttgttggacgacttgagggacgatcacccagagacaggaaaggacaggaacaaggatgcaggtaaatcaactttggtcaacaggttaggtgcagacgctgctaggcagaagttaagggagcacattgactctgctgacaagcacttgactttcgcttgcccacagggaggtgctattaggcagttcatgcacttgtggttcggtcaccacttagctgactggtcacctgtcatgaagatagcttaa

>*TmcrtE*

atgttgacttcttctaaatctattgaatcttttccaaaaaatgttcaaccatatggtaagcattatcaaaatggtttggagccagtcggtaagtctcaggaggacattttgttagagccattccactacttgtgctctaaccctggtaaggacgtcaggactaagatgatagaagcattcaatgcatggttgaaggtcccaaaggacgatttgatagttattacaagggttattgagatgttgcactctgcatcattattgattgacgacgtcgaggacgactcagtcttgaggagaggtgtccctgctgcacaccacatttacggaacaccacagactataaactgtgctaattacgtctacttcttggctttaaaggagattgctaagttaaacaaacctaacatgattactatttacacagacgagttgattaacttgcacaggggtcagggtatggagttgttctggagggacacattgacttgcccaacagagaaggagttcttggacatggtcaatgacaaaactggtggattgttgaggttggctgtcaagttgatgcaggaggcttctcagtcaggaactgactacacaggattggtttcaaagattggaatacatttccaggtcagggacgattatatgaatttacagtcaaaaaactacgcagacaacaagggattctgcgaggacttgacagagggaaagttctctttcccaataatacactcaattaggtctgacccatcaaacaggcagttgttgaacatattgaaacagagatcttcatctattgaattgaaacaattcgcattgcagttattggaaaatacaaacactttccaatattgcagagacttcttgagggtcttggagaaggaggcaagagaggaaattaaattgttgggtggtaatataatgttagaaaagattatggacgtcttgtctgtcaacgaataa

>*ScERG20*(F96C)

ATGGCTTCAGAAAAAGAAATTAGGAGAGAGAGATTCTTGAACGTTTTCCCTAAATTAGTAGAGGAATTGAACGCATCGCTTTTGGCTTACGGTATGCCTAAGGAAGCATGTGACTGGTATGCCCACTCATTGAACTACAACACTCCAGGCGGTAAGCTAAATAGAGGTTTGTCCGTTGTGGACACGTATGCTATTCTCTCCAACAAGACCGTTGAACAATTGGGGCAAGAAGAATACGAAAAGGTTGCCATTCTAGGTTGGTGCATTGAGTTGTTGCAGGCTTACTgtTTGGTCGCCGATGATATGATGGACAAGTCCATTACCAGAAGAGGCCAACCATGTTGGTACAAGGTTCCTGAAGTTGGGGAAATTGCCATCAATGACGCATTCATGTTAGAGGCTGCTATCTACAAGCTTTTGAAATCTCACTTCAGAAACGAAAAATACTACATAGATATCACCGAATTGTTCCATGAGGTCACCTTCCAAACCGAATTGGGCCAATTGATGGACTTAATCACTGCACCTGAAGACAAAGTCGACTTGAGTAAGTTCTCCCTAAAGAAGCACTCCTTCATAGTTACTTTCAAGACTGCTTACTATTCTTTCTACTTGCCTGTCGCATTGGCCATGTACGTTGCCGGTATCACGGATGAAAAGGATTTGAAACAAGCCAGAGATGTCTTGATTCCATTGGGTGAATACTTCCAAATTCAAGATGACTACTTAGACTGCTTCGGTACCCCAGAACAGATCGGTAAGATCGGTACAGATATCCAAGATAACAAATGTTCTTGGGTAATCAACAAGGCATTGGAACTTGCTTCCGCAGAACAAAGAAAGACTTTAGACGAAAATTACGGTAAGAAGGACTCAGTCGCAGAAGCCAAATGCAAAAAGATTTTCAATGACTTGAAAATTGAACAGCTATACCACGAATATGAAGAGTCTATTGCCAAGGATTTGAAGGCCAAAATTTCTCAGGTCGATGAGTCTCGTGGCTTCAAAGCTGATGTCTTAACTGCGTTCTTGAACAAAGTTTACAAGAGAAGCAAATGA

>*NaGLS*

atggactcttcttccattgtttcctctattgagtctctggtcaacgagattaagaaggacatgttctctaaccaggagttcaacacttttgtcactccaatttctgcttacgacactgcttggttggctatgatttcttataacaaccaggagaaagctactaacgaccactctttttctggtccaatgtttgagtcttgcttgaactggattttgaacaaccagaatgagcagggtttctggggtgaatctaatggtgaaaatttgccaactatctacgctttgccaactactttggcttgcatgattgctttgaaaaagtgggacgtcggtgaaactaacattaaaaagggtttgaagttcattcacgccaacactgagattttgctgaaggagaattctcagcagttcccaaggtggtttactattgtcttcccagctatggtccaattggctaaatctgttggtttggaaattatcttcgttaacggctctgagaggttgttgtctgatgtcgttatgaagaggagagttattttggagtctgagaagttgatggacgtcactgctggtcatgatcaaccattgttggcttatttggaatctttgccaaattacttcgtcgaccagaaagaccagattttgaagcacttgtctgaggacggttctttgtttcagtctccatctgctactgctcaagcttttatgtctactggtaatcagaaatgcttggagtacttgatgtctattgtccacaagtgtccaaatggtgttccatctagatttccagttgatgaggaattgattaacctgtgcatgattgaccatattcagaggatgggcttggctaaccattttaacaaggagattgagcagattttgggtcaggtttacaagaaccagatgaacaatcagaacgaatacttgtccgagatttctactttgccagagaggttgtataaggactctttggctttcaggttgttgagattgcaaggttataaaattaaccagggttctttctgctggtttgtccaccaaccacaaattagggcttatattgagaaaaaccaggagaggttcacttacgtcatgtataacgtttacagggccactgacttgatgttcaatggtgaatctgaaatggagaaggtcaggtctttcgctaggaacttcttggaatcttctatggaattggtatctaacgaggacaacttgttgttgttgccatctttgcagaaagtcattgagcacgaattgaacgttccatgggtcgctagattggaacatttggatcatagattgtggattgagctgtcccagtctttgccattgtctattggtaaatctgctgactactggttgtcttgtttgcacaatgacaaattggtcaagttggctgtccagaactacgaatacaggcagtctgtttatagaaccgagttggaagagttgaagaggtggtctaaagaaaaggacttggtcgatattggcttcggtagggagaaaactacttactcttatttcgcctctgctgcctcttcttcttcttttttgccatttgactctttcttgaggttggtcgtcgctaaatgttctattgtcattactgtcgccgacgatttttacgacgaggaggcttctttgaacgacttgaaaattttgactgacgccgtccagagatgggatggttctaatttggatggtccatctaaaattattttcgacgctctggacgacttggtctgcgatgttgctaaattgtaccatttgagacacgccattgacatcacttctgagttgaggaggcagtggcaagaaacttttttggcttggatgatggaatctacttggtctaataatgtcgctatgccatctaggaatgagtacttggaaattggtatgatttctattggcgcccacattttggtcctgcacgctgcttctttggagaatccatctttgccaagagaaaaactgaagcccattgatggtcagtacgagaatattactaagctgttgatggtcactactaggttgttgaatgacattcagtcttaccagaaggaatgcgaagttggtaagatgaactacactttgttgcatatgaacgagaacccaggtgctcaattggatgattctattgaatatgtcaaggagattttggctaacaaaaagaaggagttcttggagaacgtcttgatggacggtttcaatgacatgccaaagacttgcaagttgttgcacttgtcttgtttgaatgtcttccacatgtttttcaattcttgcaacttgttcgacactaaggctgctattttggaggacattatgagagctatttacattcccttgcaggagcaaacttctattccatctttgaagacttctcccattttgaccccacaagagaagaaaaagaagaaggagaaaaagattgagaacatccccactaaggtatctgcttgcttgaatggtaaaaacttcaagcaccaggtcggtattggtatttcttttgtcggttctaaggccccaaaaaaccatccatttggttggaatactaagggcttcgcttctcataaattgtcttcttgctttatgtga

>*AcGLS*

atggaatcttctttggctccaattcaagctttggttaatcagattaagttggagatgttctcttccactttgaacccatactctttcttgatgccatctgcttatgatactggttggttggctatgattccacatccaaaacaagattctttccccaagttcaagggctgcttggtctgggttttgaataatcagaaagaggaaggttactggggcgaaactggtactgatggtttgccaactattgattctttgccagctactttggcttgtatgatggctttgaaaaaatggggtgttggtgaaaataacgtcaagaagggtttggcttttattcacgctaattctaagatgctgttgaaggagaagtgcgaccacttgccaagatggttcactattgtttttccagccatggtcgaattggctcaagctatgaatttggaagttgtcttcccaaaagctttggaaggtgttgttttggatattttcttgaagcgccagcagattttggaaagggaggaatttgtcgaccagaactgctatccaccattggcttcttatttggaagctttgccaccatcttataacattgacaggaaagacattactatgaacttgtctggtgacggctcttttttccaatctccatctgctactgcttgtgcttttttggctactggtaatcaacaatctatggcttatttggagtctttgattcagaggtgcccaaatggtgttccagctatgtatccaattgatgccgaattgactactttgtgcttggtcaaccaaattcagaggttgggtttggctggtcatttttctgaagaagttgaagagattttgaagccaatttacaagacctacaagaaccaggaggtcgaggaaattaaaccaaatattgtccccgctaagatttacaaagactccttggctttctggttgttgaggatgtatggttacactgttactccaagaaatttctgctggttcttgtgtcacgaggacattgttgttcatattgagagaaacttcgaatgcttcacttctgctatgtataacgtctacagggctgctgacttgattttcccaggtgaatatgaattggaggaagctagattgttttccaggaaattgttggagaagtctatgaccttgaaatctgttaacgataacctggtcattttccccaacttcaggagggtcattgagcacgagttgtctttgccatggattgctagattggatcatttggatcataggatgtggattgaggagaacaaaactgacactttgtggattggtaaagcttctttttacaggttgtctaacttgcacgacgacaagttgatgactttggctgttgaaaactacaagttcaggcagtctgtctatatgaacgagttggaagagttgaaaaggtggtctaaagattggggtttgtctgacatgggttttggtagagaaaaaactacctactgctacttcgctattgcttcttctactaatttgccacacgattccattgccaggatgattgttgctaaatctgctattttggtcactgtcgctgacgatttttttgacatggaaggttctttgaacgacttgcaaactttgactcaggctgtccaaagatgggatggtgaaggtttgggtggtcatgctaaaattattttcttggctttggacaatttggtatctgaagtcgctaagaatcacttgagacaccagggtaatgatattactaagaacttgagggacatgtggattgagacttttgtctcttggttgactgagtctacttggtctaatactggttacgttccatctttggatgagtatttgcagactggtatgacttctattgccacccatattatgactttgccagctatgtgtttctctaactcttctattcacccaacttgcaagttgaacccaaatcattatgagaacattactaacttgttgatggcctctgctaggttgttgaatgacattcaatcttaccagaaggaattggaggttggcaaaaagaatttcgtcgtcttgcacttgaaagagaacccacacggtggtattgagaattctattgcttatgtcagggagttgttggacgaaatgaaaaaggaattcctggagcaggctttcatggacggtggtaatgatttgccaaaatcttttaggcacttccacttgtctattttgagggtctttcacatgttcttcaattcctctaacctgttcgactctaacaacgagttgttgcacgacattaagaaggctatttacgctccaattgagtaccagttggtcccaacttcttga

>*ScGLS*

atgactttggacgtcttgttgttttctggtttgaaattcatccacgctaacatgtacactttgctgaaggaaaactctcagtgtttcccctgctggttcactattgtttttccatctattctgcagttggccaaggctcaaggtttggaaattattattgtcaatggttctaacaagctgttgtctgacgtcattatgaagaagaaggctttgttggaatctgagaagttgaacgacaacattgttaacgaggaggacggtccattgtttcaatctccatcttctactgctcaagcttttatgtcttctggcaataagaactgcttggagtatttgatgtctatcgtccagaagtgcccaaacggtgttccatctagatttccagttgatgaagaactgaagatgttgtgcatggtcgacaacattcaaaggatgggtttggctaaacactttgagaaagagattgagcagattttggagcaggtctataataacttccagaacaacaacgaatatttgttgtcctctgaggaggagaagatttctgatgatttgccagaaaagttgttgaaggactctttggctttcaggttgttgaggttgcaaggttacaaaattaaccagggttctttctgctggtttttgcagcaaccacaaattagggataagattgagaaaaacaaggagaggttcacctacgtcatgtacaacgtctatagggctactgacttgatgtttaatggtgagtctcaaattgaggaggctaggttttttgctaggaattttttggtcaacaacaacgacaatctgtccttgttcccagctttgcaaaaggtcattgagcacgaattgaatgtcccatggtttgctagattggaacatttggatcataggctgtggattgagttgaatcagtctattcccttgtctattggcaagtcttctttctactggttgtcttacttgcagaacgagaaattgatgcagttggctgttcagaattatgagtacaggcagtctatttacaggaaggagttggaggacttgaagagatggtctaaagaaaaaggcttggtcgacattggtttcggtagggaaaaaactacttattcttacttcgcctccgccgcttcttcttcttcttttttgccatatgagtctttcttgaggttgattgtcgctaagtgttctattgtcattactgtcgctgacgacttttacgacgaggaagcttctttgtctgagttgcatattttgtctgaggctattcagaggtgggatgctaaaaatttggacggtccatctaaaatcattttcgaggctttggacgacttggtctgcgacgttgctaaaatgtatcatttgcagcacaagattgacattactccacagttgaggcatatgtggaaagaaacttttgacgcctggatgatggaatctttgtggtctagaacttctaacttgccctctagagataaatacttgcaggtcggtatgatttctattggcgctcatattttggtcttgcacgctgcttctttgggttgttctattttgccaaatcagatgttcggcccaattaacggtcaatacgaaaatgtcactaagttgttgatgactactactagattgttgaacgatattcaatcttaccagaaggagagggaggtcggtaaaatgaattatactttgttgcacttcaacgaaaacccaaggggtcaaattgaagactctattgatttcgttaaggtcattttgggtgacaagaagaaggagtttttggagaacgttttgatggacggttttaacgacatgccaagaactatgaaattgctgcacttgtcttgcttgaacgtcttccacatgttcttcaactcttgcaatttgttcgacactaagtctgctattttggaggatattatgagggctatttacatcccattgggtcagaacgaacaaactcaaattatgtccttgcaggagaagaagattaagaagaaaattgagattattaagggtaacgcttgcgtcaaccacgaaaagactatgaagcaacaggttggtattggtatttccttcgtcaaaagacagaaccccaagaactacaattctttcggttggtctactaaaggtttcgcttctcataatctgaggttgtgctttatgtaa

>*TcGLS*

atggagttgtctcatgtttctattcacgctttgattaaggagattaaggacgagatgttgtctgattatatcgacccatactctttcgtatctccatctgcttacgacactgcttggttggctatgattccagctgattctaattctcagccatgttctgctccaatgtttaaagattgtttggactgggtcttgaacaaccagactgaagaaggttattggggtgaaagagatgctcatggtaatccaactattgagtctttgccagctactttggcttgtttgattgctttgaaaaagtggaacgtcggtattgagaatgtcgaaagaggtttgactttcattcaggctaatgtcgagaagttgcataagaggaaccataacaggttcccaagatggtttactattgtcttcccagagatgattgagttcgctagaactattggtttgcagattgacttttctaaccactcccagaagttgctgattgacattttttctgagaggcagaagattttcaagattgaggagttgactgactcttcttacccattgttgttgtcttacttggaagccttgccatctttttacgctattaacgaggaagacattactatgcatttgtctgacgatggttctttgtttcagtctccatctgctactgctagagcttttatggctactggtaataaacagtgcttggcttatttgcagtctctggtcagaagatgcggtaatggtgtcccaccaacttatccaatggatgaagatttgattaagttgggcttggccaaccagttggagaggttgggtttggctgaacattttactcaacagattgaggacattttgacccaggtctacagaaactacaacaagcaggaatctttggctaaaccatctcacggtacttctattgctactcaattgtacaaagactccttggcttttaggctgttgaggatgcatggttacaatatgtctccatggtctttttgctggtttttgaataaccaggaggtcttggatcacattggtaaaaacaacgagtatttctcttctgtcatgctgaacgtctatagggctactgacttgatgttctctggtgaatatgaactggaggaggctagatctttttctaggaaggttttggaaaaggtcgcttctgagggtaatagggacgatgaccattttactaaatccttgaacttgcaaaagatgatcgagcacgagttgtctttgccatgggttgctagattggatcatttggaacatagatcctggattgaggagaacgacatgaacgctttgtgggctggtaaaacttcttttcacagattttcctctttgatgaacgaaaagttggtccagttggctgtcggtgattacgagtttagacaatctatttacaagaacgagatggctgagttgaagtcttggtgtttgaaaaggggtttgactgatatgggttttggtagagaaaaaactatgtactgctacttcgctacttctgcttgcttgtctttgccatatgattctgttattaggatgatggtcgctaagtctgctattttgattactgttgctgacgacttcttcgacatggaaggttctttgaatgagttgaacattctgactgacgctgtcaagagatgggacggtgatggtttgtctggtcatggtaaaactattttcgatgccttggacgacttggtcagggaaactgctggtaaacatttgcagcaacaaggtactgatattaagggttatttgcagcagatttggtacgagacttttgcctcttggttggttgaggctaaatggtctaaatctggttatttgccattgttggacgagtacttgggtactggtatgacttctattgctgctcatactttggttttgcccgcttcttgtttgttgaaatcttctttgccaaactctaaaatcaaccccgctgctgagtatgagactgtcactaaattggttatgctgattccaaggttgttgaacgacattcagtcttatcagaaggagattgaggacggtaaaatgaattacgtcttgttgtacatgaaggagaaccccgaagctgatattaatgactctattgcttttgtcagggacttgttggacaaaaaaaggaaggaattgctgaaacacgtcttgactgacggtttgtctgatttgccagaagcttctagacatttgcatttgtcttgtatgaaggtcttccagatgttcttcaactcttctaacaggtacgactctaacactgagatgttgcaggacattcaaaaagctatttacattccaccagacattggtatttctaagccattgatgccattgccctctgattctggtccaaaaaaagaattcaggactactacctctcacttcgtccagccagttaagtatcattctaaacgcattatcggttaccaggcttctttgccaattgctagaagaggttatgcttctatgttgattactccaaactttcgcatgtccttctcctaa

>*CaGLS*

atggactctttgccattgtcttctattgagtctttggtcattgacatgaagaaggacttgttctctaacaaggacaaggtcgctaagacttctgcttacgatactgcttggttggctatgattccaaataataacaaccaggagaacgaggacaactacggtccaatgttcgaatcttgcgttaactggattttgaacaaccagaacgagttgggtttttggggtgattttaatgtcccaactattgactgtttgccctctactttggcttgcatggttgctttgaaaaagtggggtgttggtgaatctcaaattgagaaaggtttgaagttcatccacgccaacatggacactttgctgaaggagaattctcagtgctttccaaggtcttttactattgtctttccaactattctgcagctggctaaggctcagggtttggaaattattttcattaacggttctaacaggttgttgtctgacgtctttatgaagaagaagtctattttggagtctgagaagttgaacgaccagtctgacttggcttacttggaaaagttgtctcaatctgacatttacttcaactgcgtcaacaaggacgatgagattttgaaacagttgtgcgaggaaaagaacggttctttttttcagtctccatctgctactgctcaagcttttatggctactggtaatgaaaaatgcttgcagtacttgatgtctattgtccagaagtgcccaaatggtgttccatctaggtatccaatggatgaagaattgaaaatgctgtgcaaggtcgacaacattcagaggatgggtttgggtaagcaattcgagaaagaaattggccagattttggaccaagtctacaagaactatcaggtcaacaacttggataacaacaacgacaaggaaacctcttacttgccagaaaagttgttcaaggactctttggctttcaggttgttgaggttgcaaggttataaaattaacgagggtattttctgctggttcgttcacgagccacaaattatggagtacattgagaagaaccaggacttcttcgcttatgttatgtataatgtctacagggctaccgacttgatgttcaatggtgagtctgatattgagaaggctaggtctttttctaggaatttcttggtcaacaacgacaacttgtctgtcttgccatctttgcaaaaagtcattgagcatgagttgaacgtcccatggtttgctagattggaacatttggatcataggctgtggattgagttcaaccagtctatttctttgtctgacggtaagtctccattttactggttgtcttatttgcaaaacgacaagttgactcagttggctgttcaaaactatgagtataggcagtctatttacaggaacgagttggaggatttgaagaggtggtctaaagataaaggtctggtcgacattggtttcggtagggaaaaaactacttattcttacttcgcttccgccgcttcttcttcttcttttttgccatatgagtccttcttgaggatggttgtcgctaaatgctctattgtcattactgtcgctgatgacttctacgacgaggaagcttctttgtctgaattgcaaattttgactgaggctattcagagatgggatgctaaaactttggacggtccatctaaaattattttcgaggctttggacgatttggtctgcgatgttgctaatatgtaccatttgcagcataagattgacattactccccagttgcaacacatgtggaaagagactttccactcttggatgatggagtctttgtggtctagaacttctaatttgccatgcatggatgagtacttggaggtcggtatgatttctattggtgctcatattttggtcctgcacgctgctgctttgggttgtccaactttgccaattgaaatgttgggtccaattaacggcaagtacgagaatattactaggttgttgatggctaccactaggttgttgaatgacattcagtcttaccagaaggagagggaggttggtaagatgaactatactttgttgcacttcaaggagaacccaaacggtcaaattgaggactctattgatttcgtcaagggtattgtcggtaacaagaaaaaagaattcttggagaacgtcttgatggacggttttaacgagatgccaaggactatgaaattgttgcacttgtcttgtttgaacgtcttccatatgttcttcaactcctgcaatttgttcgacactaaggctgctattttggaggatattatgagggctatttacatccccttgtctcaggaggaagagcagatttcttctatgatgccacagcaaccatctaaaactttgcagccaatggttttggaaaagaagatgaagaaagtcgagattgctaaggtcaatgcctgcttgaatgagaagaacttgaaaaagcaggtcggtattggtatttctttcgtcggtaggcaaggtccaaaatcttacaataattctttcggttggtctaacaagggctttgcttctcaaaagttgaggttgtgtttcatgtga

>GLS12

atgtctccagtcgatatctctttcattcattctgagttggtcgacagggaggaggtcgctagagtttgtgctactactttgccagttagaaaatctaagtactctccattggtcgagaaggctgttttggaatttcaacaacagtggcaagaagaggttggttttgcttattgtggtgctacttctccacaaggtccagttactgttttttttccaccagaaactaagcaggacagggtcgaaattttcgctaaattgattgagtacttcttcgcttacgacgacgtcttgactgctccaggttctgctaaaactggtgaagaacaatcttctgactccattggttgggaggttagaaagggtactacttctagagttagaatttccgccatgaagcaaattcagtctgaagtcttcttgaggttgttggagattgataggaaaaggggtaatttgattttgagggctattaacgacttgtccagggtccacggtactattggttctagagatttgagaacttgggacgatttggttcaatatagggctgatgattatggcgctgagttgaatattatgtccattatttactgctgcgagttggacttgactcaaggtgatattcaggctttgaaagagatttggtcaccagctactgctgctgctgctttggttaatgatttgtattctttcaacagggaggtcattttggagcccgatactgacactgacactactattactactccaaattctgtctggtacttgatgaagactttgaacttgtctgtctctcaggctaaagaattcttggtcaaagacaaaattttgccattggagagggagtttattgccaagaaggctgaatatttggctaacatgaatccattgactcccaagtctggtgacattatttacttcttggagatggtcggtttgggcttgtctggtaattggtattggcatgctattgctgatagattccacagatgggctgaattgttgcaattgccaccagctaaattgtttgactacgacgaggctactgctacttgtgctacttttttgaatactcagtctttgagaggtcgcaagaacaggattttggagtctactgaatctttgcaggctatgactgacgatccatattataaggttttgcaccagccaattgactacttgaggtctgttccatctaaaaacattcgtggtaccattattcaggctttgaacttgtggctgaacgccccagaatctgctgctgctcaagttgaagatttgattggtcatttgcacgagtcttctttgttgttggatgacattcaggattcctctgagttgagaagaggtaggccatctgcttatagagtttttggtgttccacaaactattaacgccgctactcatgctttgactttggcttttgaaaaagtggtcccactgatgaagccagactcttctcatgttttctttgacgagttgaggaacttgcacgttggtcaagctatggatttgtattggactagatctggttatagaccctctattgctgagtatttggagatgaataggttgaagactggtgctttgttttgcttggcttctaacttgttgtctaaccagggttctttctctgctggtgctattaaacagactgatttgaacgatttgatgatttctttgggtcagtactttcaggctagagacgattacattaacttggcctctactaagtaccaggagcagaagggttttgctcaggatttggatgagggtaaattgtctttgccattgattcacttgttgactcagtctccaaatgctgctttgattgaaaacattcagcaagagagggctaggaataataaattgccagctgatttgaagcagttgattttggacgaaatgagggaccaaaagattatgcagttgactgaagaaactttgaacggtttggaggctaaagtttatcgttacttggaaaggttggaggtatctgctggtattaagaatttcactttcaggttcctgttggacaggttgagggaaatgtaa

>*ERG9*

atgggaaagctattacaattggcattgcatccggtcgagatgaaggcagctttgaagctgaagttttgcagaacaccgctattctccatctatgatcagtccacgtctccatatctcttgcactgtttcgaactgttgaacttgacctccagatcgtttgctgctgtgatcagagagctgcatccagaattgagaaactgtgttactctcttttatttgattttaagggctttggataccatcgaagacgatatgtccatcgaacacgatttgaaaattgacttgttgcgtcacttccacgagaaattgttgttaactaaatggagtttcgacggaaatgcccccgatgtgaaggacagagccgttttgacagatttcgaatcgattcttattgaattccacaaattgaaaccagaatatcaagaagtcatcaaggagatcaccgagaaaatgggtaatggtatggccgactacatcttggatgaaaattacaacttgaatgggttgcaaaccgtccacgactacgacgtgtactgtcactacgtagctggtttggtcggtgatggtttgacccgtttgattgtcattgccaagtttgccaacgaatctttgtattctaatgagcaattgtatgaaagcatgggtcttttcctacaaaaaaccaacatcatcagagactacaatgaagatttggtcgatggtagatccttctggcccaaggaaatctggtcacaatacgctcctcagttgaaggacttcatgaaacctgaaaacgaacaactggggttggactgtataaaccacctcgtcttaaacgcattgagtcatgttatcgatgtgttgacttatttggccagtatccacgagcaatccactttccaattttgtgccattccccaagttatggccattgcaaccttggctttggtattcaacaaccgtgaagtgctacatggcaatgtaaagattcgtaagggtactacctgctatttaattttgaaatcaaggactttgcgtggctgtgtcgagatttttgactattacttacgtgatatcaaatctaaattggctgtgcaagatccaaatttcttaaaattgaacattcaaatctccaagatcgaacaattcatggaagaaatgtaccaggataaattacctcctaacgtgaagccaaatgaaactccaattttcttgaaagttaaagaaagatccagatacgatgatgaattggtcccaacccaacaagaagaagagtacaagttcaatatggttttatctatcatcttgtccgttcttcttgggttttattatatatacactttacacagagcgtga
